# DNA Methylation Analysis of Buds from Two Peach Varieties with Distinct Chilling Requirements During the Dormancy Induction Period

**DOI:** 10.3390/biology15181664

**Published:** 2026-09-20

**Authors:** Xin Jiang, Shikai Guan, Ruihong Luo, Yiwei Li, Libao Deng, Xi Wang, Yun Liu

**Affiliations:** 1 Horticulture Research Institute, Guangxi Academy of Agricultural Sciences, Nanning 530007, China; shanshan0722love@163.com (X.J.);; 2 Flower Research Institute, Guangxi Academy of Agricultural Sciences, Nanning 530007, China

**Keywords:** chilling requirement, DNA methylation, peach, *PpDAM5*, PpDAM6, whole-genome bisulfite sequencing

## Abstract

In peach production, the chilling requirement (CR) of cultivars has emerged as one of the most important limiting factors. DNA methylation plays an important role in regulating plant growth and development, environmental adaptation, and phenotypic establishment. However, the influence of DNA methylation on the CR of peach cultivars remains unclear. This study performed whole-genome methylation sequencing and transcriptome sequencing on two peach cultivars with distinct CRs and conducted an integrated analysis. The results revealed substantial differences between the two cultivars in methylation of the *PpDAM5/6* promoters, which are associated with key genes governing peach CR, and in the expression of key DNA methylation enzyme genes. These results suggest that the expression of key genes associated with CRs in peach may be affected by DNA methylation. These findings provide a new research perspective for the study of CRs in peach.

## Simple Summary

As temperate fruit trees, peaches require a sufficient accumulation of winter chilling to ensure normal flowering and fruit development. When the accumulated chilling does not satisfy the chilling requirement (CR) of a specific cultivar, substantial yield reductions or even complete crop failure may occur. Under the ongoing trend of global warming, the CRs of many conventional peach cultivars have become increasingly difficult to fulfill. This challenge is particularly pronounced in southern regions of China, where the range of suitable cultivars for cultivation is further restricted. This study aims to identify candidate methylation marks associated with differences in CRs and to explore factors that may affect the CR of peach varieties at the level of DNA methylation. The findings provide a theoretical basis for future breeding and selection of peach cultivars with suitable CRs.

## 1. Introduction

Dormancy is a phenomenon in deciduous fruit trees in which meristematic activity ceases during autumn [1]. The duration of continuous low-temperature exposure required for release from endodormancy is defined as the chilling requirement (CR). If the CR is not fulfilled, uneven flowering, reduced bud break, and decreased fruit set may occur [2,3].

CR is a quantitative trait. Rodriguez-A et al. [4] identified the non-dormant peach mutant *evergrowing (evg)*. Bielenberg et al. [5] identified six tandemly arranged MADS-box transcription factors within the EVG locus, which were not expressed in *evg* and were designated *DORMANCY ASSOCIATED MADS-box* genes (*PpDAM1* to *PpDAM6*). During dormancy, *PpDAM5* and *PpDAM6* genes exhibit high expression in high-CR cultivars and low expression in low-CR cultivars [6,7,8]. In other words, the expression levels of these two genes reflect differences in CR among cultivars. Therefore, factors regulating the expression of these two genes may also represent key determinants of CR formation in peach cultivars [9].

Numerous studies have investigated molecular networks associated with *DAMs* [3,10,11,12]. Among these studies, transcription factors [13,14] and histone modifications [15,16,17] have received greater attention. However, DNA methylation also represents an important mechanism within this regulatory network. DNA methylation involves the covalent attachment of a methyl group to the fifth carbon of cytosine in CpG dinucleotides under the action of methyltransferases. It is an evolutionarily conserved and important epigenetic modification [18,19] that can occur at CG, CHG, and CHH sites, where H represents A, T, or C, in plants [20].

DNA methylation participates in the regulation of flowering by modulating the expression of genes associated with plant vernalization and photoperiod responses [21]. FLOWERING LOCUS C (FLC) is an important transcription factor that regulates vernalization and belongs to the MADS-box family. Kondo et al. [22] found that DNA methylation regulation in Perilla is involved in *FLC* expression, and early flowering can be achieved directly through nonspecific demethylation or demethylation of methylated regions without photoperiodic induction. Williams et al. [23] found that *DME* demethylase mutant plants of Arabidopsis thaliana exhibited an early-flowering phenotype, which was associated with 5′-terminal hypermethylation and transcriptional downregulation of *FLC*.

Several key DNA methylation regulators functionally analogous to DME have been identified in plant systems, including the maintenance methyltransferase DNA METHYLTRANSFERASE 1 (MET1) [24], the plant-specific CHROMOMETHYLASE 3 (CMT3) [25], the de novo methyltransferase DOMAINS REARRANGED METHYLTRANSFERASE 2 (DRM2) [18,26], and the chromatin remodelling factor DECREASED DNA METHYLATION 1 (DDM1) [27,28]. Collectively, these proteins govern the accurate establishment and faithful propagation of genomic DNA methylation patterns across cell divisions. Complementing this methylation machinery are active DNA demethylases, most prominently DEMETER (DME) [29] and REPRESSOR OF SILENCING 1 (ROS1) [30], which mediate targeted removal of methyl marks to shape transcriptionally permissive chromatin states.

The key genes associated with peach CR, *PpDAM5* and *PpDAM6*, also belong to the *MADS-box* family. However, few studies have reported the regulation of *DAM* gene expression by DNA methylation in peach. Zhu et al. [17] comprehensively examined changes in three DNA methylation contexts among six peach *DAM* genes. Canton et al. [15] also investigated DNA methylation changes in *DAM* genes in peach buds under different chilling accumulations. However, these studies did not compare differences among cultivars with distinct CRs. Furthermore, the regulatory relationship between the expression of *PpDAM5* and *PpDAM6* and the aforementioned key enzymes governing methylation regulation remains poorly characterized.

In this study, whole-genome bisulfite sequencing (WGBS) and transcriptome sequencing (RNA-seq) were employed to examine buds from two peach cultivars with distinct CRs during the dormancy induction period. DNA methylation patterns and target-gene expression were analyzed. DNA methylation at the *PpDAM5* and *PpDAM6* gene loci was examined specifically, and inter-cultivar differences were compared to infer whether DNA methylation status is associated with target-gene expression. These results refine the epigenetic regulatory module in the *DAM* genes’ regulatory network. This information lays a theoretical foundation for the future breeding and selection of peach cultivars based on CRs, with a focus on the *PpDAM5* and *PpDAM6* genes.

## 2. Materials and Methods

### 2.1. Plant Materials and Treatments

Buds from 3-year-old trees of the peach cultivars ‘Xiahui No. 8’ (CR 768 h) and ‘Yingzui’ (CR 174 h), which had been planted in Dongli Village, Bala Township, Tian’e County, Guangxi, China (25° N, 107° E), were sampled at four time points during the dormancy induction period. The CR was measured using the 0–7.2 °C model. The sampling time points were 1 November, 15 November, 29 November, and 13 December 2024. Bud samples were collected, rapidly frozen in liquid nitrogen, and stored at −80 °C. Three biological replicates were collected for each sample.

### 2.2. Whole-Genome Bisulfite Sequencing Analysis

Genomic DNA from peach buds was extracted using a DNeasy Plant Maxi Kit (Qiagen, Hilden, Germany) according to the manufacturer’s protocol. Each sample had three biological replicates.

After genomic DNA was extracted from the samples, DNA concentration and integrity were assessed using a NanoDrop spectrophotometer (Thermo, Waltham, MA, USA) and agarose gel electrophoresis, respectively. Genomic DNA was fragmented into 100–300 bp fragments by sonication (Covaris, Woburn, MA, USA) and purified using a MiniElute PCR Purification Kit (Qiagen, Hilden, Germany). The fragmented DNA was end-repaired, and a single “A” nucleotide was added to the 3′ end of the blunt fragments. Subsequently, the genomic fragments were ligated to methylated sequencing adapters. Finally, the converted DNA fragments were polymerase chain reaction (PCR)-amplified and sequenced using NovaSeq X Plus by Gene Denovo Biotechnology Co. (Guangzhou, China).

The obtained clean reads were mapped to the species reference genome (Prunus_persica_NCBIv2, https://www.ncbi.nlm.nih.gov/datasets/genome/GCF_000346465.2 (accessed on 13 March 2025)) using BSMAP software [31] (version: 2.90) with default parameters. Then, a custom Perl script was used to call methylated cytosines, and the methylated cytosines were tested using the correction algorithm described by Lister et al. [32]. Due to the limitations of objective factors, SNP correction was not conducted.

Differential DNA methylation between the two samples at each locus was determined using Pearson’s chi-square test (χ^2^) in the methylKit package1.26.0 [33]. To identify differentially methylated cytosines (DMCs) and differentially methylated regions (DMRs), the minimum read coverage required to determine the methylation status of a base was set to 4. DMCs for each sequence context (CG, CHG, and CHH) between the two samples were identified according to different criteria: for CG and CHG, the absolute difference in the methylation ratio was ≥0.25, with q ≤ 0.05; for CHH, the absolute difference in the methylation ratio was ≥0.15, with q ≤ 0.05; and for all C, the absolute difference in the methylation ratio was ≥0.2, with q ≤ 0.05. DMRs for each sequence context (CG, CHG, and CHH) were identified according to different criteria. For CG and CHG, the number of CGs in each window was ≥5; for CHH, the number of CHHs in each window was ≥15; and for all C, the number of Cs in each window was ≥20.

### 2.3. RNA Extraction, Library Construction, and Sequencing

Total RNA was extracted using an RNAprep Pure Plant kit for polysaccharide- and polyphenolic-rich samples (Tiangen Biotech, Beijing, China) and then enriched after rRNA removal using a Ribo-ZeroTM Magnetic kit (Epicentre, Madison, WI, USA). Both procedures were performed according to the manufacturers’ instructions. Extraction was conducted in triplicate.

An Illumina TruSeq TM RNA Sample Preparation kit (Illumina, San Diego, CA, USA) was used to construct the RNA-seq cDNA libraries. Then, the Illumina NovaSeq 6000 sequencing system (Shanghai Biozeron Co., Ltd., Shanghai, China) was used to sequence the paired-end libraries. Trimmomatic v.0.36 was used with the ILLUMINACLIP:adapters.fa:2:30:10, SLIDINGWINDOW:4:15, and MINLEN:75 parameters (http://www.usadellab.org/cms/index.php?page=trimmomatic (accessed on 26 January 2025)) were used to trim the raw paired-end reads for quality control [34]. To obtain high-quality clean reads, reads were further filtered using fastp software v. 0.18.0 [35]. The clean reads were then aligned to the reference genome, the same genome used for WGBS, using HISAT v.2. 0.5 with default parameters [36]. FeatureCounts software v1.6.3 and the fragments per kilobase of exon per million mapped reads method were used to determine read counts and relative expression levels for each gene.

### 2.4. qRT-PCR Analysis

The specific primer pairs used for qRT-PCR are listed in Table S1. Total bud RNA was extracted using the same method described in the section above. ChamQTM Universal SYBR^®^ qPCR Master Mix (Vazyme, Nanjing, China), a QTOWER 2.2 fluorescence quantitative gradient PCR instrument (Analytik Jena, Jena, Germany), and a fluorescence image analysis system (Tanon 5200 Multi, Tanon, Shanghai, China) were used to perform the qRT-PCR analysis according to the manufacturers’ instructions. The 2^−ΔΔCt^ method was used to calculate the relative expression levels of the genes [37], and the reference gene was the *P. persica Actin* gene (LOC18779708).

### 2.5. Statistical Analysis of the Data

All of the gene expression measurements were repeated three times, and the data were analyzed using Excel 2013 (Microsoft Corp., Redmond, WA, USA) and SPSS 20.0 (IBM, Armonk, NY, USA). The data are expressed as the mean ± SD. One-way analysis of variance (significance threshold *p* < 0.05) was used to test differences in gene expression between cultivars; Pearson correlation tests were used to assess correlations between gene expression and methylation level; differential DNA methylation between the two samples at each locus was determined using Pearson’s chi-square test (χ^2^) in methylKit [33]; and DEGs were defined as unigenes with a false discovery rate < 0.001 and a fold change > 2.

### 2.6. Prediction of Transcription Factor Binding Sites

The promoter sequences of *PpDAM5* and *PpDAM6* were downloaded from the NCBI database (NCBI accession numbers of all the genes in this study were shown in Table S2), and the JASPAR online tool (https://jaspar.elixir.no/ (accessed on 9 November 2023)) was subsequently applied to identify the core binding motifs of PpCBF and PpABF transcription factors on these two promoters.

## 3. Results

### 3.1. Differences in DNA Methylation Between the Two Cultivars

To investigate the relationship between DNA methylation and the CR of peach cultivars, DNA methylation in peach buds from two cultivars with markedly different CRs was examined by WGBS during the dormancy induction period. Temporal changes in DNA methylation levels were assessed at four time points before leaf fall. The WGBS conversion rates were >98%.

**Figure 1:**
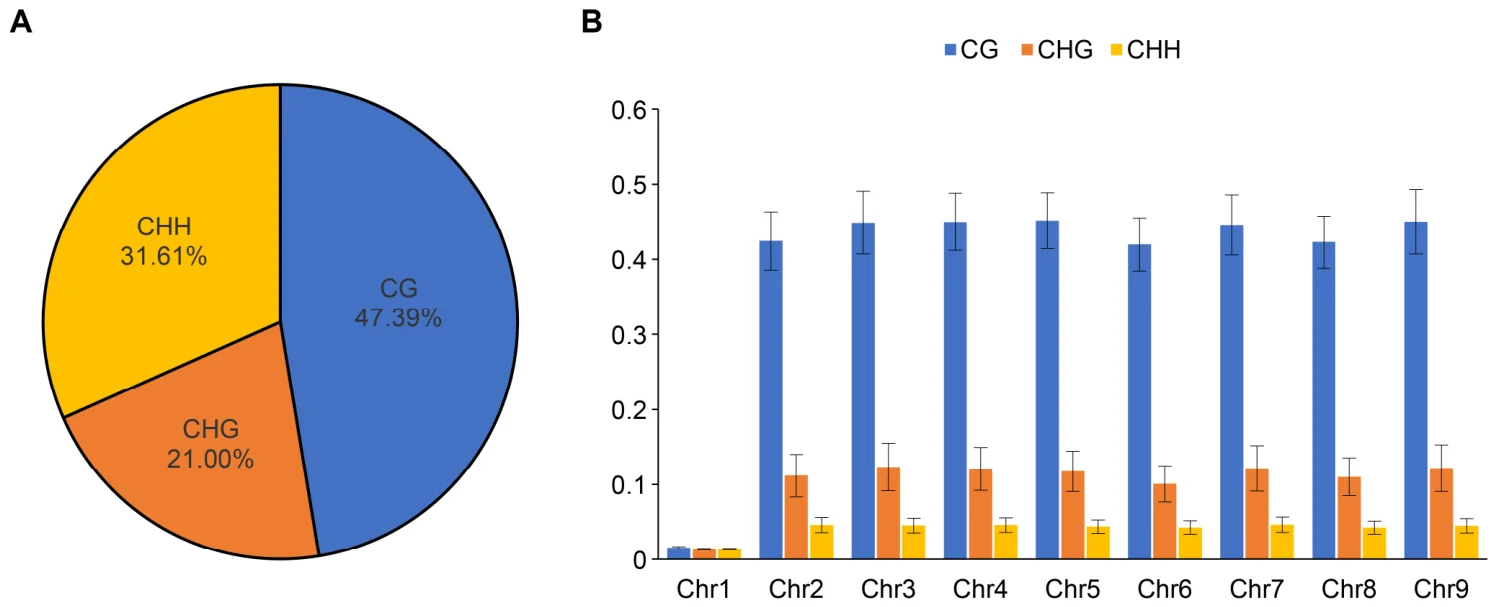
Overall methylation characteristics of peach buds. (**A**) Relative proportion of mCs in the three sequence contexts, CG, CHG, and CHH, in peach. (**B**) Average methylation level of 24 samples on each chromosome under the three sequence contexts, CG, CHG, and CHH.

The distribution of DNA methylation across three sequence contexts, CG, CHG, and CHH, together with gene density, is presented in Figure 1. Based on genome-wide statistics for methylation-site distribution, CG contexts, accounting for 47.39%, were the major contributors to methylation sites in the whole genome of peach buds (Figure 1A). Figure 1B shows the average methylation level in the three contexts, i.e., CG, CHG, and CHH, on each chromosome.

**Figure 2:**
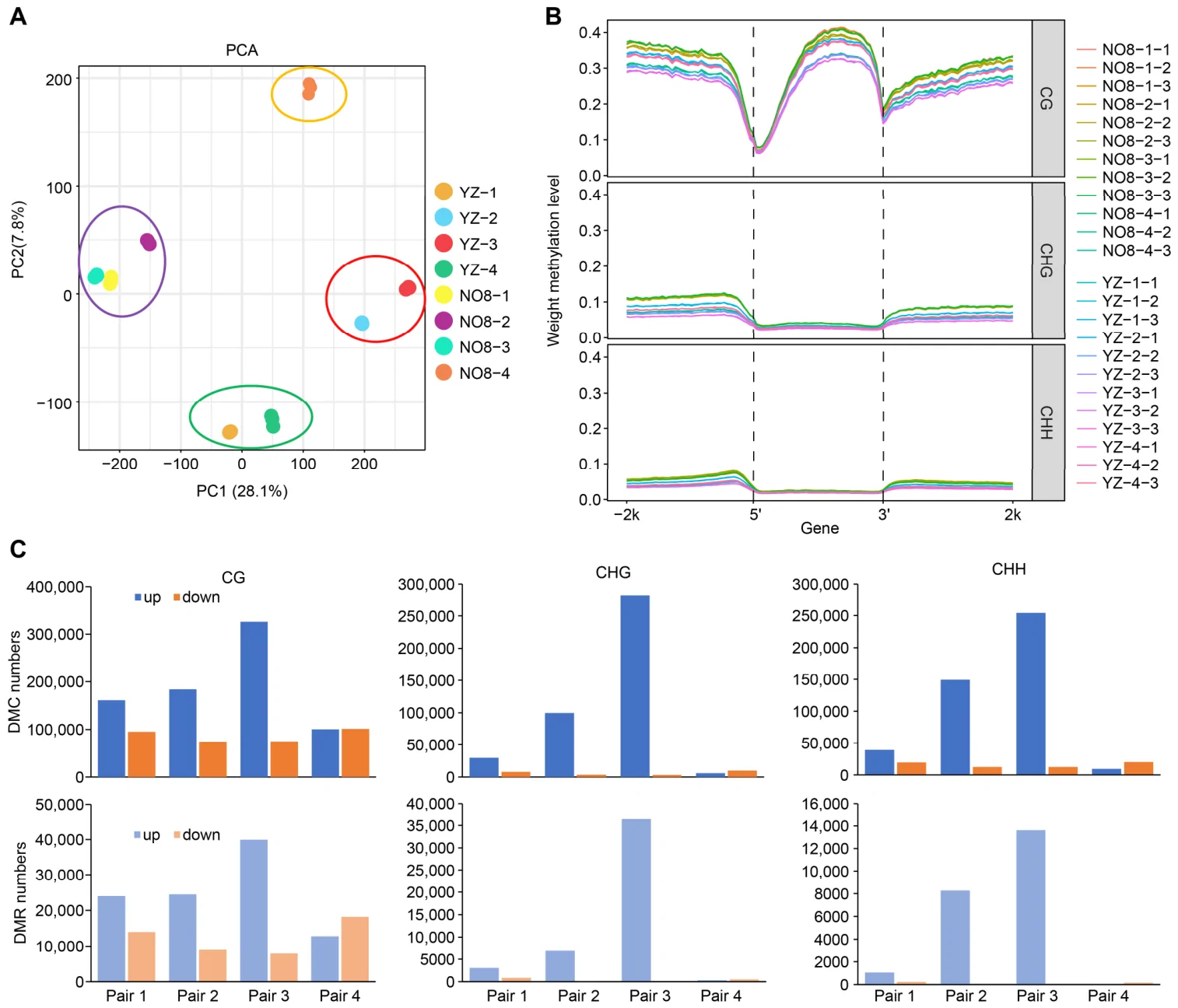
Comparative analysis of methylation during the dormancy induction period in peach buds from two cultivars. (**A**) Principal component analysis of DMRs. (**B**) DNA methylation levels of genes at different time points in the two cultivars (Three biological replicates for each sample were showed). (**C**) Differences in the numbers of DMCs and DMRs between ‘Yingzui’ and ‘Xiahui No. 8’ under the mCG, mCHG, and mCHH sequence contexts. Each analysis was repeated three times.

PCA analysis showed that genes associated with differentially methylated regions (DMRs) were classified into four groups (Figure 2A). The DMRs from the first and fourth time points of ‘Yingzui’ peach were grouped together, whereas those from the second and third time points were grouped together. The DMRs from the fourth time point of ‘Xiahui No. 8’ were grouped separately, whereas those from the other three time points were grouped together.

DNA methylation levels in gene bodies and in the 5′ and 3′ flanking regions of genes were then analyzed, revealing significant differences between the two cultivars, with ‘Xiahui No. 8’ showing higher levels than ‘Yingzui’ (Figure 2B). Notably, the first time point of ‘Xiahui No. 8’ exhibited the highest DNA methylation level, whereas the final time point of ‘Yingzui’ showed the lowest level (Figure 2B).

A total of 4,149,436 differentially methylated cytosines (DMCs) and 227,746 differentially methylated regions (DMRs) were identified between ‘Yingzui’ and ‘Xiahui No. 8’. Because SNP correction was not performed during the analysis, these numbers may be overestimated relative to the true level of methylation variation. Compared with ‘Xiahui No. 8’, ‘Yingzui’ had 176,409 hypermethylated DMRs (hyper-DMRs) and 51,337 hypomethylated DMRs (hypo-DMRs). This means that among the regions with differential methylation, those exhibiting methylation gains in ‘Yingzui’ were more prevalent than those exhibiting methylation losses. This predominance of hyper-DMRs reflects the direction of methylation changes in individual genomic regions and the observation that ‘Xiahui No. 8’ exhibited a higher average methylation level across gene bodies and their flanking regions. In other words, although most DMRs corresponded to localized methylation increases in ‘Yingzui’, the overall methylation landscape remained higher in ‘Xiahui No. 8’. Notably, the third sampling time point was associated with the largest difference in DMR abundance between the two cultivars (Figure 2C).

**Figure 3:**
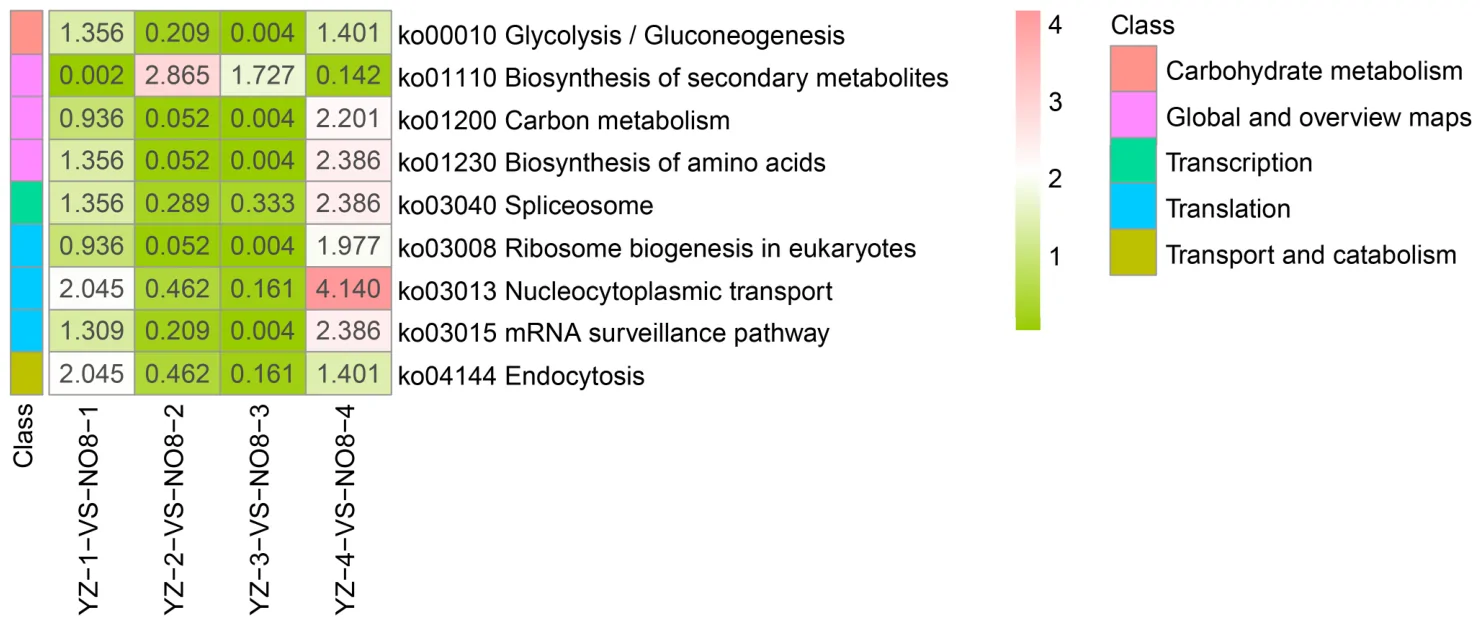
KO enrichment results of DMR-related genes (−log_10_ (Q) value).

Differential enrichment analysis was conducted for all of the methylation sites. In both the Gene Ontology (GO) and Kyoto Encyclopedia of Genes and Genomes (KO) enrichment results, numerous differences in methylation levels between the two cultivars were associated with transcription-related processes, including the mRNA surveillance pathway (ko03015) in the KO enrichment results (Figure 3) and DNA replication (GO:0006260), RNA processing (GO:0006396), RNA modification (GO:0009451), and mRNA metabolic process (GO:0016071) in the GO enrichment results (Figure S1). The difference was greatest at the fourth time point, indicating that the difference in DNA methylation levels between the two cultivars reached its maximum when dormancy began.

### 3.2. Analysis of Methylation Levels at the PpDAM5 and PpDAM6 Gene Loci

**Figure 4:**
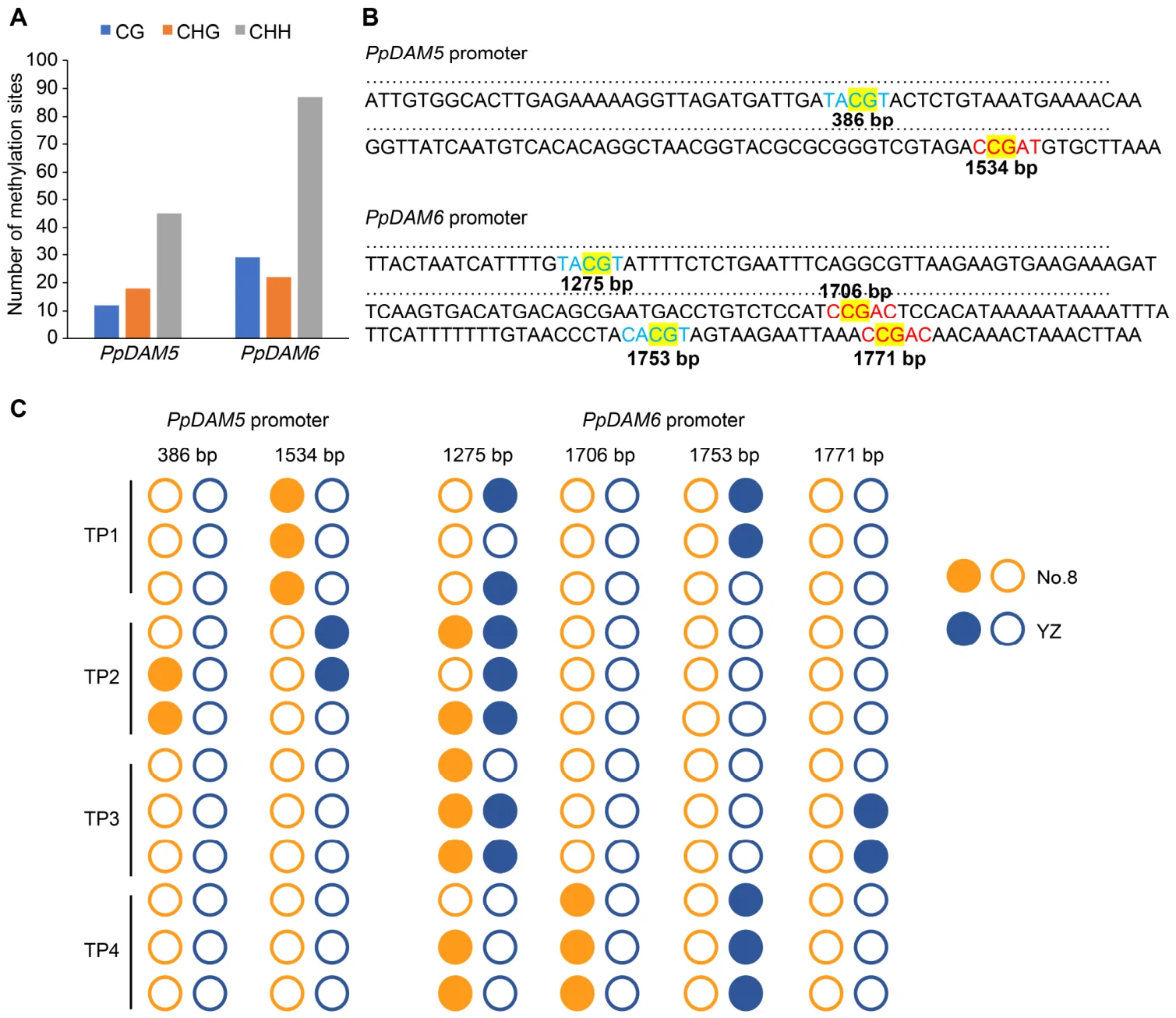
Methylation analysis of the *PpDAM5* and *PpDAM6* gene promoters. (**A**) Number of the three types of methylation sites in the gene promoter regions. (**B**) Transcription factor binding sites, with blue indicating ABF binding sites and red indicating CBF binding sites. Yellow highlights indicate methylation sites. (**C**) Methylation of transcription factor binding sites, with the solid circle indicating methylation and hollow circles indicating no methylation. TP stands for time point.

Given the significant differences in genetic background between the two cultivars, the methylation status of the *PpDAM5* and *PpDAM6* gene loci was specifically analyzed to clarify the relationship between DNA methylation and cultivar CR. In total, 411 methylation sites were detected in the *PpDAM5* gene body, and 75 sites were detected in its promoter region. In total, 397 methylation sites were detected in the *PpDAM6* gene body, and 138 sites were detected in its promoter region. The numbers of the three types of methylation sites in the promoter regions of the two genes are shown in Figure 4A.

The 386 bp and 1534 bp sites of the *PpDAM5* gene promoter are both binding sites for the CBF transcription factor. The 1706 bp and 1771 bp sites of the *PpDAM6* gene promoter are also binding sites for the CBF transcription factor, whereas the 1275 bp and 1753 bp sites are binding sites for the ABF transcription factor (Figure 4B). The combined core motifs of the CBF and ABF transcription factors were analyzed from the promoter sequences of *PpDAM5* and *PpDAM6* using JASPAR online software. Both types of transcription factors have been reported to regulate the expression of *PpDAM5* and *PpDAM6*. Further comparison of the methylation of these sites revealed differences between the two cultivars (Figure 4C).

### 3.3. Differences in the Transcriptome of Peach Buds Between the Two Cultivars During the Dormancy Induction Period

To compare changes in gene expression induced by dormancy in buds of peach cultivars with different CRs, transcriptome analysis was performed. A total of 24 RNA-seq libraries were used for sequencing, generating 141.8 Gb of clean reads (Table S3).

**Figure 5:**
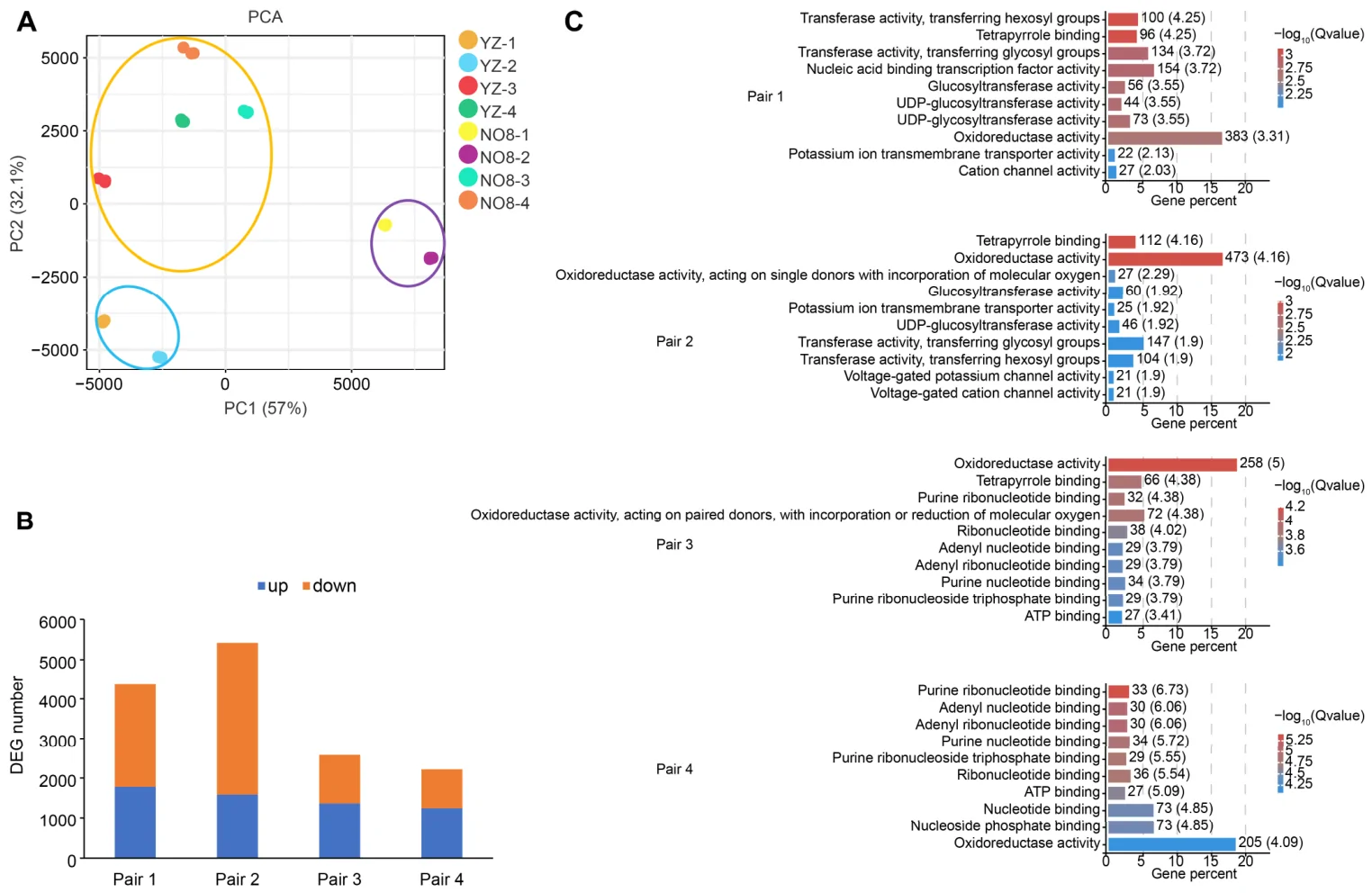
Comparison of transcriptome sequencing results from peach buds of two cultivars during the dormancy induction period. (**A**) PCA results of RNA-seq. (**B**) DEG numbers between the two cultivars. (**C**) Top 10 GO enrichment terms.

Compared with ‘Xiahui No. 8’ peach, 14,581 differentially expressed genes (DEGs) were identified across four time points in ‘Yingzui’ peach. According to expression patterns during the dormancy induction process, the PCA model divided these DEGs into three groups (Figure 5A). The DEGs of ‘Yingzui’ peach at the first two time points clustered into one group. The DEGs of ‘Xiahui No. 8’ peach at the first two time points clustered into the second group, whereas the DEGs of the two cultivars at the final two time points clustered into the third group. As dormancy induction progressed, the number of DEGs gradually decreased (Figure 5B). This result indicated that a significant difference existed between the two cultivars at the early stage of dormancy induction and that the difference gradually decreased as the dormancy period approached. Notably, throughout the process, the number of upregulated genes in ‘Yingzui’ peach changed only slightly compared with ‘Xiahui No. 8’ peach, whereas the number of downregulated genes decreased markedly. This pattern indicates that, at the early stage of dormancy induction, gene expression in ‘Yingzui’ peach was more strongly inhibited, which is consistent with the larger number of hyper-DMRs detected in the methylation results for ‘Yingzui’ peach.

Enrichment analysis was conducted for DEGs. The top 10 GO enrichment results were mainly related to transcriptional activity and nucleic acid binding (Figure 5C), indicating that transcriptional regulation of gene expression is the main source of the difference in gene expression between the two cultivars during the dormancy induction period. This finding is also consistent with the enrichment results of DMR-related genes.

### 3.4. Influence of DNA Methylation on Gene Expression

**Figure 6:**
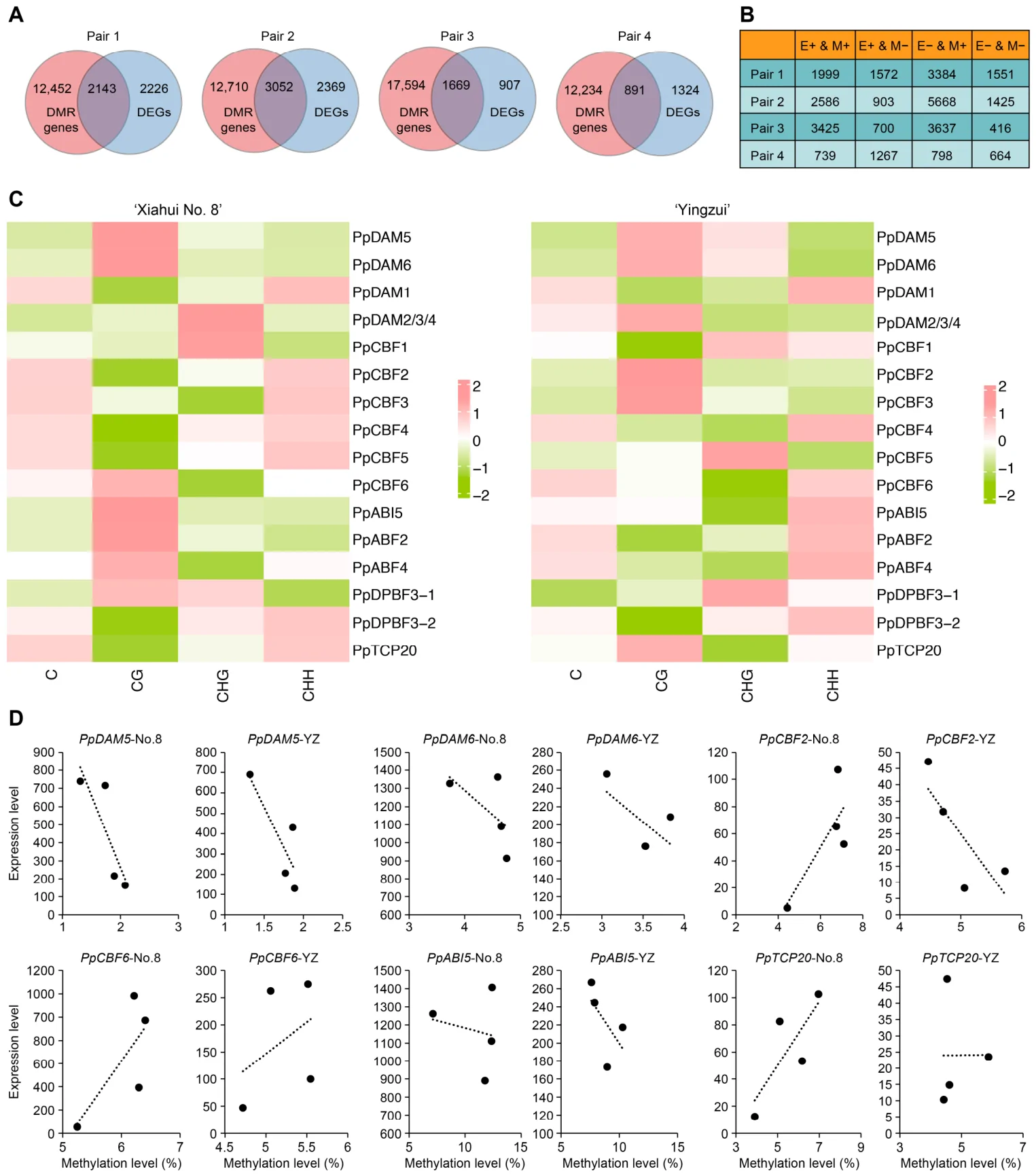
Correlation between DNA methylation and gene expression levels in peach buds during dormancy induction. (**A**) Venn plot showing the overlap between total DMR-related genes and total DEGs. (**B**) Number of genes with different DMR and DEG trend combinations. (**C**) Heatmap showing the correlation between DNA methylation in promoter regions and expression levels of genes related to chilling requirement. (**D**) Correlations between DNA methylation in promoter regions and gene expression levels of PpDAM5, *PpDAM6, PpCBF2*, *PpCBF6*, *PpABI5*, and *PpTCP20*.

The influence of DNA methylation on the expression of DMR-related genes during dormancy induction in peach buds was analyzed. The number of DMR-related genes showing differential expression at each time point is presented in Figure 6A. In addition, differentially methylated high- and low-methylated genes and differentially expressed up- and downregulated genes in peach buds during dormancy induction were compared. For example, according to the RNA-seq data, 3384 genes with hypermethylated DMRs showed downregulated expression in peach buds at the first time point (Figure 6B). A much lower percentage was observed for the fraction of genes shared between hypo-DMGs and downregulated DEGs. For example, only 416 hypo-DMGs were downregulated in peach buds at the third time point (Figure 6B). Most reports indicate that DNA methylation inhibits gene expression, and the present result was consistent with this pattern.

The correlation between transcript abundance and DNA methylation was then examined for genes related to CR. *PpDAM5* and *PpDAM6* have been reported as key genes controlling the CR of peach cultivars. The correlation analysis showed that none of these correlations were statistically significant. However, the possible relationships between them can still be explored based on the similarity of their change trends. The expression trends of *PpDAM5* and *PpDAM6* were contrary to the methylation trends at their promoter sites in both cultivars (Figure 6C,D). PpCBFs, PpABFs, and PpTCP20 are transcription factors reported to exert regulatory effects on *DAM* gene expression. Among them, PpCBF2, PpCBF6, PpABI5, and PpTCP20 have been demonstrated to bind to the promoters of *PpDAM5* and *PpDAM6* and regulate their expression. Except for *PpCBF6*, the change trends in methylation levels and transcript levels observed in the other genes differed between the two cultivars (Figure 6C,D). The expression trend of *PpCBF6* was similar to the methylation trend in its promoter in both cultivars. In ‘Xiahui No. 8’, the expression trend of *PpCBF2* was similar to its promoter methylation trend, whereas in ‘Yingzui,’ the opposite pattern was observed (Figure 6D). These results indicate that different members of the same transcription factor family may perform distinct regulatory functions.

### 3.5. Genes Regulating DNA Methylation

DNA methylation regulates gene expression, and, correspondingly, specific genes also regulate DNA methylation.

**Figure 7:**
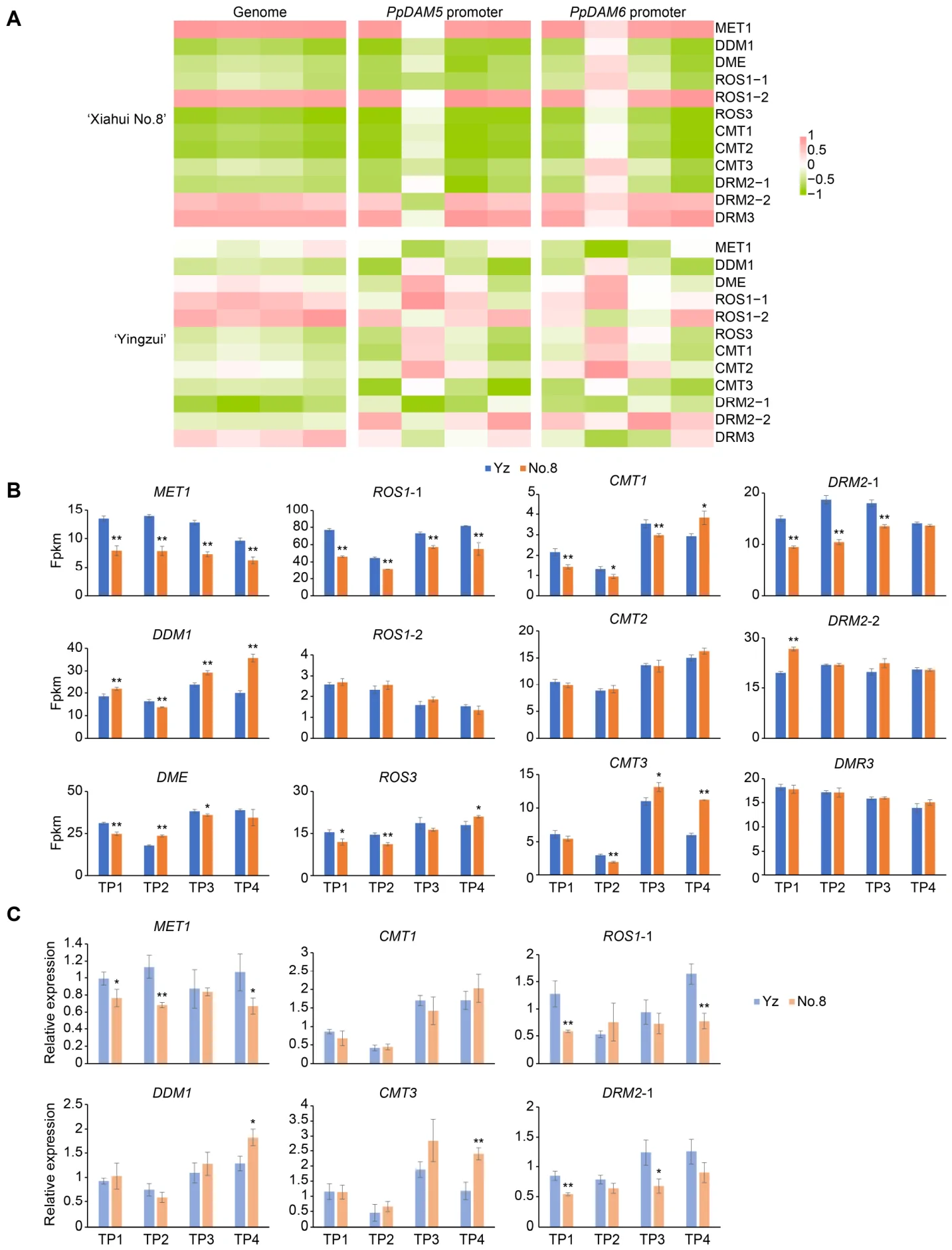
Genes regulating DNA methylation. (**A**) Correlations between DNA methylation key enzyme gene expression levels and DNA methylation levels. (**B**) Comparison of DNA methylation key enzyme gene expression levels between the two cultivars based on RNA-seq. (**C**) Comparison of DNA methylation key enzyme gene expression levels between the two cultivars based on qRT-PCR. * indecated *p* < 0.05. ** indecated *p* < 0.01.

The results revealed differences between ‘Xiahui No. 8’ and ‘Yingzui’ in the correlations between the expression of DNA methylation-related enzyme genes and DNA methylation levels. For example, *MET1* expression and DNA methylation level showed a positive correlation in ‘Xiahui No. 8’, whereas in ‘Yingzui,’ they primarily exhibited a negative correlation (Figure 7A). In ‘Xiahui No. 8,’ the correlations between the expression of each gene and the methylation levels of the *PpDAM5/6* promoters were generally consistent with the correlations between gene expression and genomic methylation levels, except at CG sites (Figure 7A). However, a markedly different pattern was observed in ‘Yingzui.’

From the perspective of correlations with genomic methylation levels, the expressions of *MET1*, *ROS1-2*, *DRM2-2*, and *DRM3* were positively correlated in ‘Xiahui No. 8’, whereas *ROS1-1*, *ROS1-2*, and *DRM3* showed positive correlations in ‘Yingzui’ (Figure 7A). The correlations between gene expression and methylation at CG sites in the *PpDAM5/6* promoter regions differed substantially from those observed for the other two site types. In ‘Xiahui No. 8,’ the genes positively correlated with CG-site methylation in the *PpDAM6* promoter region were *MET1*, *DME*, *ROS1-1*, *CMT3*, and *DRM2-2* (Figure 7A), whereas these genes showed negative correlations with CG-site methylation in the *PpDAM5* promoter. In ‘Yingzui,’ the correlations of CG-site methylation in the promoter regions of *PpDAM5* with each DNA methylation-regulatory gene were relatively consistent with those of *PpDAM6*. Among them, the genes showing positive correlations with DNA methylation were *DME*, *ROS1-1*, *ROS3*, *CMT1*, and *CMT2* (Figure 7A).

The expression differences in these key enzyme genes involved in DNA methylation were further compared between the two cultivars, and *MET1*, *DDM1*, *CMT1*, *CMT3*, *ROS1*-1, and *DRM2*-1 showed significant differences (Figure 7B). This difference was further validated using quantitative reverse transcription–polymerase chain reaction (qRT-PCR), and the results were generally consistent with those obtained from RNA-seq (Figure 7C). The differential expression between the two cultivars observed via qRT-PCR assay is less pronounced than that revealed by RNA-seq analysis. The significance of *MET1* expression difference is relatively consistent across the two determination methods.

## 4. Discussion

### 4.1. DNA Methylation Differences Among Peach Cultivars with Different CRs

Studies have shown that epigenetic modifications play significant roles in regulating plant growth and development. These modifications can cause variation in plant gene expression or phenotype without altering the DNA sequence and can also be inherited [38]. DNA methylation is inherited mitotically and meiotically [39,40] and plays distinct roles across different sequence contexts and gene regions [41]. Therefore, in this study, the significant differences in genomic DNA methylation between the two peach cultivars, ‘Xiahui No. 8’ and ‘Yingzui,’ which have different genetic backgrounds, are reasonable. The results of this study show that there were significant differences between the two varieties, both in their overall methylation levels and in the methylation of individual gene loci (Figure 2 and Figure 4).

DNA methylation regulates gene expression by recruiting proteins involved in gene repression or activation or by affecting the binding of inhibitory or activating transcription factors to DNA [42]. The differential enrichment results for DNA methylation (Figure 3 and Figure S1) and transcriptomes (Figure 5C) in the two cultivars indicated that many significantly different GO and KO terms were associated with DNA and protein binding.

However, DNA methylation occurs throughout all of the processes of plant growth and development [41,43]. In addition to the significant difference in CRs, the two cultivars mentioned above also differ substantially in other characteristics, such as tree architecture, fruit traits, and related phenotypic aspects. Differences in overall genomic DNA methylation do not directly indicate that DNA methylation is one of the sources of the differences in CRs between the two cultivars. Therefore, this study specifically analyzed DNA methylation of *PpDAM5* and *PpDAM6*, which are key genes for CR, as well as other related regulatory genes.

The results showed significant differences in DNA methylation in the promoter regions of *PpDAM5* and *PpDAM6* among peach cultivars with different CRs, particularly at the binding sites of several transcription factors that have been demonstrated to regulate their expression (Figure 4). Zhu et al. [17] conducted a relatively comprehensive analysis of DNA methylation and the expression of six *DAM* genes in peach. However, comparisons among cultivars with different CRs were not performed in their study, and no other related reports have been published. Therefore, this study conducts, for the first time, a comparative analysis of DNA methylation in *DAM* gene promoters among peach cultivars with different CRs. However, this work was conducted for only one winter and at one location. In the future, additional test sites and sampling times could be included to further validate the results and enhance the scientific rigor of the study.

In addition, since SNP correction was not performed in this study, the total numbers of DMCs and DMRs were very likely overestimated. Therefore, whether this affected the differences in methylation of the *PpDAM5* and *PpDAM6* gene promoters that were of particular concern in this study could be further analyzed. As shown in Figure 4C, the methylation levels at individual sites within the same cultivar showed temporal differences under the same genetic background. Therefore, these methylation differences are likely to be real.

### 4.2. Key Enzyme Genes Regulating DNA Methylation in Peach Buds During the Dormancy Induction Period

CG methylation in plants is mainly catalyzed by MET1 [24]. The methylation of CHG and CHH sites mainly depends on CMT3 [25] and DRM2 [18,26]. All three types of DNA methylation are related to DDM1 [27,28]. DME [29] and ROS1 [30] can catalyze the DNA demethylation process in plants. However, very few studies have examined these enzymes in fruit trees, and no related research reports have been found in peach.

In this study, multiple key enzyme genes associated with DNA methylation were identified from the WGBS and RNA-seq results, including *MET1*, *DDM1*, *CMT1*, *CMT3*, *ROS1*-1, and *DRM2*-1, which differed significantly between the two cultivars (Figure 7). Li et al. [44] showed that overexpression of *CmDRM2a* from Chrysanthemum morifolium can significantly increase the CHH methylation level in the promoter region of *CmMYB6*, a key anthocyanin-regulatory gene, thereby inhibiting its expression and leading to reduced anthocyanin synthesis and lighter flower color. Meanwhile, overexpression of *CmDRM2b* and *CmCMT2* also enhanced the CHH methylation level in the promoter region of *CmMYB6* but promoted *CmMYB6* expression, intensified anthocyanin accumulation, and deepened flower color. Therefore, this study speculates that the DNA methylation level of peach can also be regulated by modulating the expression of key DNA methylation enzyme genes. Specifically, if these genes regulate DNA methylation in the promoters of *PpDAM5* and *PpDAM6* and thereby regulate their expression, the CR of the cultivar may be modulated. This regulatory relationship requires further experimental verification, such as through genetic transformation of the above-mentioned key genes or the use of DNA methylation inhibitors. If this regulatory relationship is verified through these experiments, regulating the CR of peach cultivars through DNA methylation may represent a potential direction in breeding low-CR cultivars.

## 5. Conclusions

The results of this study indicated that significant differences in DNA methylation exist between the two peach cultivars with distinct CRs during the dormancy induction period, particularly in the promoter regions of the key CR genes *PpDAM5* and *PpDAM6* and at the binding sites of related transcription factors. These methylation differences might represent candidate methylation marks associated with differences in CRs. These results provide a new perspective for future CR selection breeding in peach.

## Data Availability

The raw WGBS and RNA-seq sequence data generated in this study were deposited in the Genome Sequence Archive. WGBS: CRA029387, https://ngdc.cncb.ac.cn/gsa/browse/CRA029387 (accessed on 15 September 2026), RNA-seq: CRA030159, https://ngdc.cncb.ac.cn/gsa/browse/CRA030159 (accessed on 15 September 2026).

## Supplementary Materials

The following supporting information can be downloaded at: https://www.mdpi.com/article/10.3390/biology15181664/s1, Figure S1: GO Enrichment Results of DMC-related genes (*p* value); Table S1. List of primers used in qRT-PCR. Table S2. List of NCBI accession numbers for core genes in this study. Table S3. Summary of RNA-Seq reads mapping results.
