# Supplementary material for: DNA Methylation Analysis of Buds from Two Peach Varieties with Distinct Chilling Requirements During the Dormancy Induction Period
Source: Biology (Basel). 2026 Sep 20;15(18):1664. doi: 10.3390/biology15181664 (PMC13604107; doi:10.3390/biology15181664)
Supplement: Supplementary file 1 [file biology-15-01664-s001.zip › biology-4516851-supplementary.pdf]

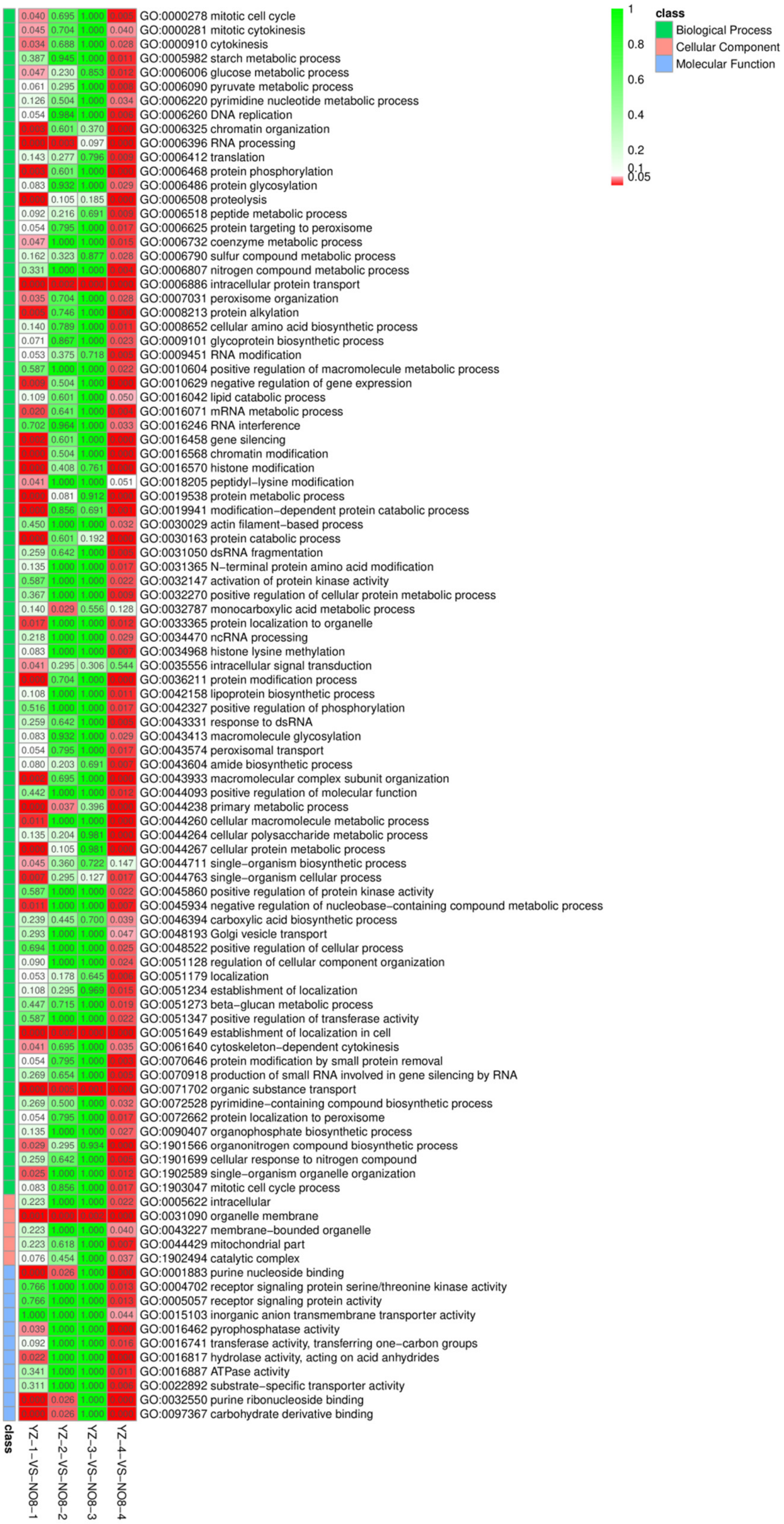
GO:0000278 mitotic cell cycle
GO:0000281 mitotic cytokinesis
GO:0000910 cytokinesis
GO:0005982 starch metabolic process
GO:0006006 glucose metabolic process
GO:0006090 pyruvate metabolic process
GO:0006220 pyrimidine nucleotide metabolic process
GO:0006260 DNA replication
GO:0006325 chromatin organization
GO:0006396 RNA processing
GO:0006412 translation
GO:0006468 protein phosphorylation
GO:0006486 protein glycosylation
GO:0006508 proteolysis
GO:0006518 peptide metabolic process
GO:0006625 protein targeting to peroxisome
GO:0006732 coenzyme metabolic process
GO:0006790 sulfur compound metabolic process
GO:0006807 nitrogen compound metabolic process
GO:0006886 intracellular protein transport
GO:0007031 peroxisome organization
GO:0008213 protein alkylation
GO:0008652 cellular amino acid biosynthetic process
GO:0009101 glycoprotein biosynthetic process
GO:0009451 RNA modification
GO:0010604 positive regulation of macromolecule metabolic process
GO:0010629 negative regulation of gene expression
GO:0016042 lipid catabolic process
GO:0016071 mRNA metabolic process
GO:0016246 RNA interference
GO:0016458 gene silencing
GO:0016568 chromatin modification
GO:0016570 histone modification
GO:0018205 peptidyl−lysine modification
GO:0019538 protein metabolic process
GO:0019941 modification−dependent protein catabolic process
GO:0030029 actin filament−based process
GO:0030163 protein catabolic process
GO:0031050 dsRNA fragmentation
GO:0031365 N−terminal protein amino acid modification
GO:0032147 activation of protein kinase activity
GO:0032270 positive regulation of cellular protein metabolic process
GO:0032787 monocarboxylic acid metabolic process
GO:0033365 protein localization to organelle
GO:0034470 ncRNA processing
GO:0034968 histone lysine methylation
GO:0035556 intracellular signal transduction
GO:0036211 protein modification process
GO:0042158 lipoprotein biosynthetic process
GO:0042327 positive regulation of phosphorylation
GO:0043331 response to dsRNA
GO:0043413 macromolecule glycosylation
GO:0043574 peroxisomal transport
GO:0043604 amide biosynthetic process
GO:0043933 macromolecular complex subunit organization
GO:0044093 positive regulation of molecular function
GO:0044238 primary metabolic process
GO:0044260 cellular macromolecule metabolic process
GO:0044264 cellular polysaccharide metabolic process
GO:0044267 cellular protein metabolic process
GO:0044711 single−organism biosynthetic process
GO:0044763 single−organism cellular process
GO:0045860 positive regulation of protein kinase activity
GO:0045934 negative regulation of nucleobase−containing compound metabolic process
GO:0046394 carboxylic acid biosynthetic process
GO:0048193 Golgi vesicle transport
GO:0048522 positive regulation of cellular process
GO:0051128 regulation of cellular component organization
GO:0051179 localization
GO:0051234 establishment of localization
GO:0051273 beta−glucan metabolic process
GO:0051347 positive regulation of transferase activity
GO:0051649 establishment of localization in cell
GO:0061640 cytoskeleton−dependent cytokinesis
GO:0070646 protein modification by small protein removal
GO:0070918 production of small RNA involved in gene silencing by RNA
GO:0071702 organic substance transport
GO:0072528 pyrimidine−containing compound biosynthetic process
GO:0072662 protein localization to peroxisome
GO:0090407 organophosphate biosynthetic process
GO:1901566 organonitrogen compound biosynthetic process
GO:1901699 cellular response to nitrogen compound
GO:1902589 single−organism organelle organization
GO:1903047 mitotic cell cycle process
GO:0005622 intracellular
GO:0031090 organelle membrane
GO:0043227 membrane−bounded organelle
GO:0044429 mitochondrial part
GO:1902494 catalytic complex
GO:0001883 purine nucleoside binding
GO:0004702 receptor signaling protein serine/threonine kinase activity
GO:0005057 receptor signaling protein activity
GO:0015103 inorganic anion transmembrane transporter activity
GO:0016462 pyrophosphatase activity
GO:0016741 transferase activity, transferring one−carbon groups
GO:0016817 hydrolase activity, acting on acid anhydrides
GO:0016887 ATPase activity
GO:0022892 substrate−specific transporter activity
GO:0032550 purine ribonucleoside binding
GO:0097367 carbohydrate derivative binding
class
YZ−1−VS−NO8−1
YZ−2−VS−NO8−2
YZ−3−VS−NO8−3
YZ−4−VS−NO8−4
1
0.8
0.6
0.4
0.2
0.1
0.05
class
Biological Process
Cellular Component
Molecular Function

Figure S1. GO Enrichment Results of DMC-related genes (*P* value).

Table S1. List of primers used in qRT-PCR.

Gene	Primer	Sequence (5' to 3')
Actin	*Actin* F	CAGATCATGTTTGAGACCTTCAATGT
	Actin R	CATCACCAGAGTCCAGCACAAT
MET1	*MET1* F	GCTTCGGTGGCAAGTTACAA
	MET1 R	GCTCTTACATTGGCTAGGTCAG
DDM1	*DDM1* F	CAACTTCGGAAGAACTGCTGTC
	DDM1 R	TGCCACACTGCTCAACCATC
CMT1	*CMT1* F	AGGCTACAACAACAAGGCTGAT
	CMT1 R	CTTCTTCAACTTCCTCCTCCTCTT
CMT3	*CMT3* F	CTCCATCCAATTCCGCTTCAG
	*CMT3*R	GCTTCAGGCTCCTCTCCATC
ROS1-1	*ROS1*-1 F	AAGAAGAGCGTAGAGTGTTCCA
	ROS1-1R	CCAACCACTGAATCCACAACTG
DRM2-1	*DRM2*-1F	AGTGACTTTCTCTCTCTAGA
	DRM2-1R	GAGAGCTCCGATCATCCG

Table S2. List of NCBI accession numbers for core genes in this study.

Gene	NCBI accession number
PpDAM5	LOC18793896
PpDAM6	LOC18789329
PpDAM1	LOC18792812
PpDAM2/3/4	LOC18790353
PpCBF1	LOC18778067
PpCBF2	LOC18776669
PpCBF3	LOC18776409
PpCBF4	LOC18776400
PpCBF5	LOC18777414
PpCBF6	LOC18787317
PpABI5	LOC18770072
PpABF2	LOC18791667
PpABF4	LOC18766233
PpDPBF3-1	LOC18787415
PpDPBF3-2	LOC18774771
PpTCP20	LOC18781726
CMT1	LOC18771792
CMT2	LOC18777830
CMT3	LOC18773528
DRM2-1	LOC18766166
DRM2-2	LOC18782160

DRM3	LOC18791468
MET1	LOC18773295
DDM1	LOC18769108
DME	LOC18770234
ROS1-1	LOC18771193
ROS1-2	LOC18774609
ROS3	LOC18789872

Table S3. Summary of RNA-Seq reads mapping results.

Sample	Total	Unmapped (%)	Unique_Mapped (%)	Multiple_Mapped(%)	Total_Mapped (%)
YZ-1-1	35093120	1925591 (5.49%)	32489437 (92.58%)	678092 (1.93%)	33167529 (94.51%)
YZ-1-2	34722124	1895267 (5.46%)	32155502 (92.61%)	671355 (1.93%)	32826857 (94.54%)
YZ-1-3	35352102	2048370 (5.79%)	32625739 (92.29%)	677993 (1.92%)	33303732 (94.21%)
YZ-2-1	43684958	2415949 (5.53%)	40424434 (92.54%)	844575 (1.93%)	41269009 (94.47%)
YZ-2-2	38674080	2117917 (5.48%)	35815383 (92.61%)	740780 (1.92%)	36556163 (94.52%)
YZ-2-3	34736314	2010713 (5.79%)	32057504 (92.29%)	668097 (1.92%)	32725601 (94.21%)
YZ-3-1	35334310	2429092 (6.87%)	32233673 (91.22%)	671545 (1.90%)	32905218 (93.13%)
YZ-3-2	43263012	3139356 (7.26%)	39280650 (90.79%)	843006 (1.95%)	40123656 (92.74%)
YZ-3-3	38410922	2863560 (7.46%)	34802295 (90.61%)	745067 (1.94%)	35547362 (92.54%)
YZ-4-1	35132438	2302653 (6.55%)	32191740 (91.63%)	638045 (1.82%)	32829785 (93.45%)
YZ-4-2	38752904	2333506 (6.02%)	35707388 (92.14%)	712010 (1.84%)	36419398 (93.98%)
YZ-4-3	42166918	2509437 (5.95%)	38875363 (92.19%)	782118 (1.85%)	39657481 (94.05%)
NO8-1-1	41020256	4117646 (10.04%)	36253666 (88.38%)	648944 (1.58%)	36902610 (89.96%)
NO8-1-2	46786658	4758817 (10.17%)	41291661 (88.26%)	736180 (1.57%)	42027841 (89.83%)

NO8-1-3	45954876	4620541 (10.05%)	40614651 (88.38%)	719684 (1.57%)	41334335 (89.95%)
NO8-2-1	44852282	3526561 (7.86%)	40573876 (90.46%)	751845 (1.68%)	41325721 (92.14%)
NO8-2-2	42554782	3550739 (8.34%)	38299948 (90.00%)	704095 (1.65%)	39004043 (91.66%)
NO8-2-3	43835278	3566194 (8.14%)	39547921 (90.22%)	721163 (1.65%)	40269084 (91.86%)
NO8-3-1	37580650	2410209 (6.41%)	34495357 (91.79%)	675084 (1.80%)	35170441 (93.59%)
NO8-3-2	42080088	2752888 (6.54%)	38580192 (91.68%)	747008 (1.78%)	39327200 (93.46%)
NO8-3-3	38938404	2552692 (6.56%)	35687250 (91.65%)	698462 (1.79%)	36385712 (93.44%)
NO8-4-1	36580192	2172916 (5.94%)	33720415 (92.18%)	686861 (1.88%)	34407276 (94.06%)
NO8-4-2	36662912	2189664 (5.97%)	33759493 (92.08%)	713755 (1.95%)	34473248 (94.03%)
NO8-4-3	40922354	2398312 (5.86%)	37749564 (92.25%)	774478 (1.89%)	38524042 (94.14%)
